# Side Effects of Endocrine Therapy Are Associated With Depression and Anxiety in Breast Cancer Patients Accepting Endocrine Therapy: A Cross-Sectional Study in China

**DOI:** 10.3389/fpsyg.2022.905459

**Published:** 2022-05-09

**Authors:** Rong Zhao, Hulin Liu, Jinnan Gao

**Affiliations:** Department of Breast Surgery, Shanxi Bethune Hospital, Tongji Shanxi Hospital, Shanxi Academy of Medical Sciences, Third Hospital of Shanxi Medical University, Taiyuan, China

**Keywords:** anxiety, breast cancer, depression, psycho-oncology, side effects

## Abstract

**Objective:**

Hormone positive breast cancer patients bear side effects of endocrine therapy and that may be related to depression and anxiety. We sought to find an association between mental health and side effects of endocrine therapy.

**Methods:**

A total of 398 patients participated. Sociodemographic, disease profile, and side effects questionnaires were administered. We screened for depressive and anxiety disorders by using the SDS (Self-Rating Depression Scale) and SAS (Self-Rating Anxiety Scale).

**Results:**

The prevalence of depression and anxiety in our study were 33.4% (133) and 13.3% (53), respectively. Depression was linked to education level (≤8 years, OR = 3.59, 95% CI: 2.22–5.78), night sweats (yes, OR = 1.90, 95% CI: 1.17–3.09), vaginal dryness (yes, OR = 2.22, 95% CI: 1.19–4.16), and fatigue (yes, OR = 1.94, 95% CI: 1.21–3.11); anxiety was associated with education level (≤8 years, OR = 3.13, 95% CI: 1.62–6.08), time to diagnosis (≤ 3 years, OR = 2.14, 95% CI: 1.13–4.07), osteopenia (yes, OR = 2.43, 95% CI: 1.26–4.70), loss of hair (yes, OR = 2.80, 95% CI: 1.10–7.15), and fatigue (yes, OR = 2.89, 95% CI: 1.54–5.43). A stratified analysis according to age (≤45 years and > 45 years) was performed as an exploratory. None of factor-age interactions was statistically significant.

**Conclusion:**

Side effects of endocrine therapy were significantly associated with anxiety and depression. Side effects deserve greater emphasis and clinical interventions are needed to reduce anxiety and depression in breast cancer patients accepting ET.

## Introduction

Depression and anxiety are very common among breast cancer patients, which could lead to poor adherence to the treatment, and is associated with substantial functional impairment and increased risk of mortality (Pitman et al., 2018; Shim et al., 2020). However, there is not enough attention given to the psychological conditions of the breast cancer survivors in clinical practice for several reasons, such as insufficient medical resources. Identification of patients at high risk of depression and/or anxiety is needed.

Apart from body image issues (Woertman and van den Brink, 2012), identity construction (McGannon et al., 2016), social and intimate relationships (Sebri et al., 2021), which have been confirmed relate to breast cancer patients’ mood disorders by the literature, cancer patients’ tumor characteristics, treatment, sociodemographic characteristics, such as marital status, educational status, and age, may affect psychologic status to a large extent (Stark et al., 2002; Osborne et al., 2003; Linden et al., 2012; Tsaras et al., 2018). However, the conclusion from previous studies have been discrepant. For example, educational status was a significant predictive factor in some studies (Tsaras et al., 2018), while it did not exhibit significant differences in other studies. In addition, the prevalence of depression and anxiety varies widely among studies (Krebber et al., 2014; Tsaras et al., 2018; Hashemi et al., 2020), indicating the prevalence varies between different individuals is large. Breast cancer patients accepting endocrine therapy (ET) is a particular population.

Endocrine therapy is a standard treatment for hormone positive (HR+) breast cancer patients (Noordhoek et al., 2021). The widespread used medicine includes tamoxifen, a selective estrogen-receptor modular (SERM), and aromatase inhibitor (AI), such as letrozole, anastrozole or exemestane. The standard treatment period lasts for 5 years. However, a recent study showed extending adjuvant ET for 10 years linked to a survival benefit for selected women (Noordhoek et al., 2021). Beyond this, some subgroup patients could benefit from ovarian function suppression (OFS) (Nagaraj and Ma, 2021; Noordhoek et al., 2021). As a strategy of breast cancer therapy, ET is associated with the side effect, especially when the intensity of the medication increases. Estrogens therapies could lead to estrogen deprivation and have widespread toxicity, involving multiple systems. The side effect of ET could impair patients’ quality of life and is associated with depression and anxiety (Condorelli and Vaz-Luis, 2018).

We aimed to investigate the rate of depression/anxiety in breast cancer patients accepting ET and the relationship between side effects of ET and mood disorders, recognize the most commonly observed side effect related to depression and anxiety, and generate basic data. Therefore, this study may contribute evidence to support screening for depression/anxiety and integrating it into treatment.

## Materials and Methods

Ethical approval was obtained through the Shanxi Bethune Hospital Ethics Committee. Patients with breast cancer who had visited Shanxi Bethune Hospital between March 2012 and December 2017 were recruited. Female patients were included in the study if they met the following inclusion criteria: (1) aged 18 or older; (2) had undergone breast surgery and were undergoing adjuvant endocrine therapy; (3) could read, understand, and write in mandarin; and (4) could be connected by telephone. (1) Who was at the disease progression stage; (2) was diagnosed the pre-existing mental illness; or (3) those who did not want to participate in the study were excluded. This resulted in a final sample size of 398 patients.

A coordinator nurse recruited eligible patients and explained the study to them by phone. Once oral consent had been received from the patients, an electronic informed consent would send to them by WeChat, and the participant provided an electronic signature. Then the data was collected immediately by phone, including demographic, disease-specific information (e.g., surgery and menopausal status), side effects of ET, depression and anxiety. Depression and anxiety were evaluated by scale.

### Side Effects of Endocrine Therapy

Estrogens therapies could impact the reproductive, musculoskeletal, cardiovascular, and central nervous systems. The main clinical manifestations are vaginal irritation, dryness, arthralgia, osteopenia, osteoporosis, bone fractures, hypercholesterolemia, angina, myocardial, hot flashes, night sweats, fatigue, and headache (Condorelli and Vaz-Luis, 2018). The nurse ticked the box according to the patients’ answers. The detailed meaning would be explained by the nurse if necessary.

### Self-Rating Depression Scale and Self-Rating Anxiety Scale

For assessment of the patient’s subjective view of symptoms, we used the SDS (Self-Rating Depression Scale) (Zung, 1965) and SAS (Self-Rating Anxiety Scale) (Zung, 1971). The SDS has a split-half reliability of 0.73 (Dunstan et al., 2017) and is with an alpha coefficient of 0.68–0.81 (Knight et al., 1983; Tanaka-Matsumi and Kameoka, 1986; DeForge and Sobal, 1988). Reported correlations with other depression scales include 0.41 with the Hamilton Rating Scale (Carroll et al., 1973), 0.54 with the Depression Adjective Checklist, and 0.68 with the Beck Depression Inventory (Tanaka-Matsumi and Kameoka, 1986). The SAS has been shown to have good internal consistency with a Cronbach’s alpha of 0.82 (Tanaka-Matsumi and Kameoka, 1986); fair concurrent validity, correlating significantly (0.30) with the Taylor Manifest Anxiety Scale (Zung, 1971). The SDS and SAS both contain 20 items and their designs were based on the diagnostic criteria for depression and anxiety. Subjects scored each item according to how they have felt during the past several days using a 4-point Likert scale. The raw sum score of the SDS and SAS ranges from 20 to 80 but results are usually presented as the SDS or SAS Index, which is converted by the raw score to 100 points scale. We used 50 points on the SDS and SAS index score for cutoff value (Dunstan and Scott, 2019, 2020).

### Statistical Analysis

All data were analyzed through Statistical Package for Social Sciences (SPSS) V.26. Missing values were imputed using 20-fold multiple imputation. Categorical variables are reported as a proportion (%), and numerical data are reported as median and corresponding 25th and 75th percentiles (interquartile range; IQR) or range. Univariate and multivariate logistic analysis were performed for statistical analysis and the odds ratios (OR) and their 95% confidence intervals (CI) were calculated. In the multivariate regression model, the Stepwise Forward method was used. In addition, a stratified analysis was performed as an exploratory, adjunct analysis. Factor-age interactions were evaluated by logistic model including an interaction term between factors and age. All *p*-values were considered statistically significant at two-tailed *p* < 0.05.

## Results

### Demographic and Disease-Related Characteristics

A total of 398 patients were analyzed in our study. Participants’ median age was 51 years (range: 24–92 years); 29.1% (116) were 45 years or younger; a total of 35.9% (143) lived in urban area; 25.6% (102) were working; 50.8% (202) accepted education less than 8 years; 94.5% (376) were married; and 39.7% (158) participants with cessation of menses for > 1 year. Most patients (312, 78.4%) without comorbidity and 44% (175) accepted breast-conserving surgery. The majority of patients conducted chemotherapy (314, 78.9%) and radiotherapy (273, 68.6%), and only 9% (36) conducted HER2-targeted therapy. Most patients were stage 1/2, accounting for 76.8% (306). At the time of the investigation, 52.0% (207) were diagnosed less than 3 years and 35.2% (140) were treated with ET less than 1 year. 61.6% (245) patients accepted AI and the others accepted tamoxifen or toremifene (153, 38.4%).

About one-third of patients suffered from depression (133, 33.4%) and one of seven suffered from anxiety (53, 13.3%). 35.7% (142) patients suffered from depression or anxiety, and 11.1% (44) patients suffered from depression and anxiety. The demographic and disease-related characteristics distribution in the depression group and anxiety group were similar to the total population. 4.3% (17) patients in our study seek help from a psychiatrist, and the rates were higher in the depression group (7, 5.3%) and anxiety group (3, 5.7%). Over a half of patients (226, 56.8%) seek help from their relatives. Patients suffering from depression (77, 57.9%) tended to seek relatives’ help than patients suffering from anxiety (24, 45.3%) (Table 1).

**TABLE 1:** Demographic, disease-related characteristics, and side effects in total populations and depression/anxiety groups.

	Total	Depression	Anxiety
*N*	398 (100.0%)	133 (33.4%)	53 (13.3%)
Age at follow-up (median, range)	51 (24–92)	50 (26–88)	51 (29–92)
≤45 years	116 (29.1%)	43 (32.3%)	12 (22.6%)
>45 years	282 (70.9%)	90 (67.7%)	41 (77.4%)
Place of residence			
Urban	143 (35.9%)	50 (37.6%)	16 (30.2%)
Rural	255 (64.1%)	83 (62.4%)	37 (69.8%)
Employment status			
Working	102 (25.6%)	34 (25.6%)	10 (18.9%)
Retired	60 (15.1%)	19 (14.3%)	8 (15.1%)
Not working	236 (59.3%)	80 (60.2%)	35 (66.0%)
Education level			
≤8 years	202 (50.8%)	89 (66.9%)	38 (71.7%)
>8 years	196 (49.2%)	44 (33.1%)	15 (28.3%)
Marital status			
Divorced, widowed, single	22 (5.5%)	9 (6.8%)	7 (13.2%)
Married	376 (94.5%)	124 (93.2%)	46 (86.8%)
Menstrual status			
Cessation of menses for ≤ 1 year	240 (60.3%)	85 (63.9%)	35 (66.0%)
Cessation of menses for > 1 year	158 (39.7%)	48 (36.1%)	18 (34.0%)
Comorbidity			
Yes	86 (21.6%)	31 (23.3%)	12 (22.6%)
No	312 (78.4%)	102 (76.7%)	41 (77.4%)
Surgery			
BCS^†^	175 (44.0%)	61 (45.9%)	25 (47.2%)
Mastectomy	223 (56.0%)	72 (54.1%)	28 (52.8%)
Chemotherapy	314 (78.9%)	105 (78.9%)	36 (67.9%)
HER2-targeted therapy	36 (9.0%)	13 (9.8%)	3 (5.7%)
Radiotherapy	273 (68.6%)	99 (74.4%)	40 (75.5%)
TNM stage			
0	10 (2.5%)	6 (4.5%)	1 (1.9%)
1	116 (29.1%)	41 (30.8%)	19 (35.8%)
2	190 (47.7%)	66 (49.6%)	25 (47.2%)
3/4	82 (20.6%)	20 (15.0%)	8 (15.1%)
Time to diagnosis (median, IQR^‡^)	34.6 (20.7–52.5)	31.5 (18.1–52.9)	26.5 (14.2–55.4)
≤36 m	207 (52.0%)	74 (55.6%)	33 (62.3%)
>36 m	191 (48.0%)	59(44.4%)	20 (37.7%)
ET^§^ duration (median, IQR)	24.0 (12.0–36.0)	20.0 (9.3–42.0)	18.0 (9.0–48.5)
≤12 m	140 (35.2%)	50 (37.6%)	20 (37.7%)
>12 m	258 (64.8%)	83 (62.4%)	33 (62.3%)
ET medicine			
Tamoxifen/toremifene	153 (38.4%)	53 (39.8%)	25 (47.2%)
AI^¶^	245 (61.6%)	80 (60.2%)	28 (52.8%)
Side effects			
Night sweats	130 (32.7%)	53 (39.8%)	22 (41.5%)
Facial flushing	47 (11.8%)	18 (13.5%)	6 (11.3%)
Vaginal bleeding	10 (2.5%)	2 (1.5%)	2 (3.8%)
Vaginal dryness	54 (13.6%)	26 (19.5%)	9 (17.0%)
Sexual dysfunction	59 (14.8%)	26 (19.5%)	10 (18.9%)
Venous thrombosis	12 (3.0%)	5 (3.8%)	2 (3.8%)
Endometrial thickening	39 (9.8%)	18 (13.5%)	3 (5.7%)
Eye toxicity	6 (1.5%)	3 (2.3%)	1 (1.9%)
Hypertension	35 (8.8%)	16 (12.0%)	5 (9.4%)
Dyslipidemia	19 (4.8%)	8 (6.0%)	2 (3.8%)
Musculoskeletal symptoms	131 (32.9%)	51 (38.3%)	24 (45.3%)
Osteopenia	89 (22.4%)	37 (27.8%)	20 (37.7%)
Weight gain	99 (24.9%)	39 (29.3%)	14 (26.4%)
Loss of hair	30 (7.5%)	12 (9.0%)	8 (15.1%)
Loss of appetite	32 (8.0%)	15 (11.3%)	8 (15.1%)
Fatigue	123 (30.9%)	55 (41.4%)	27 (50.9%)
Seek care from a psychiatrist	17 (4.3%)	7 (5.3%)	3 (5.7%)
Seek care from relatives	226 (56.8%)	77 (57.9%)	24 (45.3%)
Pharmacotherapy	37 (9.3%)	16 (12.0%)	7 (13.2%)

*^†^Breast-conserving surgery; ^‡^interquartile range; ^§^endocrine therapy; ^¶^aromatase inhibitor.*

### Side Effects of Patients During Endocrine Therapy

Side effects of ET included night sweats (130, 32.7%), facial flushing (47, 11.8%), vaginal bleeding (10, 2.5%), vaginal dryness (54, 13.6%), sexual dysfunction (59, 14.8%), venous thrombosis (12, 3.0%), endometrial thickening (39, 9.8%), eye toxicity (6, 1.5%), hypertension (35, 8.8%), dyslipidemia (19, 4.8%), musculoskeletal symptoms (131, 32.9%), osteopenia (89, 22.4%), weight gain (99, 24.9%), loss of hair (30, 7.5%), loss of appetite (32, 8.0%), and fatigue (123, 30.9%). Most proportions of positive symptoms in the two groups were similar to the total population.

The most common discomforts for patients with depression or anxiety were night sweat (39.8% in the depression group and 41.5% in the anxiety group, respectively), musculoskeletal symptoms (38.3 and 45.3%, respectively), and fatigue (41.4 and 50.9%, respectively) (Table 1).

### Univariate Analysis of Depression and Anxiety

The statistically significant factors for depression included education level (≤8 years, OR = 2.72, 95% CI: 1.76–4.21), night sweats (yes, OR = 1.62, 95% CI: 1.05–2.50), vaginal dryness (yes, OR = 2.06, 95% CI: 1.15-3.68), and fatigue (yes, OR = 2.04, 95% CI: 1.31–3.18). Significant factors for anxiety included education level (≤8 years, OR = 2.80, 95% CI: 1.48–5.27), marital status (divorced, widowed, or single, OR = 3.35, 95% CI: 1.30–8.65), chemotherapy (yes, OR = 1.97, 95% CI: 1.01–3.83), musculoskeletal symptoms (yes, OR = 1.84, 95% CI: 1.02–3.31), osteopenia (yes, OR = 2.42, 95% CI: 1.31–4.48), loss of hair (yes, OR = 2.61, 95% CI: 1.10–6.21), loss of appetite (yes, OR = 2.38, 95% CI: 1.01–5.61), and fatigue (yes, OR = 2.69, 95% CI: 1.50–4.85) (Table 2).

**TABLE 2:** Univariate analysis of depression and anxiety.

	Depression		Anxiety	
	cOR	95% CI	cOR	95% CI
Age (years, vs. >45)	1.26	0.80–1.98	0.68	0.34–1.34
Place of residence (vs. rural)	1.04	0.67–1.62	0.69	0.37–1.32
Employment Status (vs. not working)				
Working	1.00	0.61–1.64	0.66	0.31–1.39
Retired	0.91	0.49–1.70	0.78	0.32–1.86
Education level (years, vs. >8)	2.72*	1.76–4.21	2.80*	1.48–5.27
Marital status (vs. married)	1.41	0.59–3.38	3.35*	1.30–8.65
Menstrual status (vs. > 1 year)	0.91	0.59–1.40	0.89	0.49–1.63
Comorbidity (vs. no)	0.94	0.57–1.55	1.10	0.52–2.30
Surgery (vs. mastectomy)	1.12	0.73–1.70	1.16	0.65–2.08
Chemotherapy (vs. yes)	1.12	0.69–2.00	1.97*	1.01–3.83
HER2-targeted therapy (vs. yes)	0.96	0.41–2.23	2.25	0.56–9.03
Radiotherapy (vs. yes)	0.84	0.53–1.31	0.67	0.33–1.37
Stage (stage 2/3/4 vs. stage 0/1)	1.18	0.74–1.88	1.30	0.71–2.41
Time to diagnosis (vs. 36 m)	1.24	0.81–1.88	1.61	0.89–2.92
ET^†^ duration (vs. >12 m)	1.17	0.76–1.81	1.12	0.62–2.04
ET medicine (vs. AI^‡^)	1.09	0.71–1.67	1.51	0.84–2.70
Side effects				
Night sweats (vs. no)	1.62*	1.05–2.50	1.56	0.86–2.82
Facial flushing (vs. no)	1.27	0.68–2.39	0.95	0.38–2.35
Vaginal bleeding (vs. no)	0.49	0.10–2.34	1.65	0.34–8.00
Vaginal dryness (vs. no)	2.06*	1.15–3.68	1.36	0.62–2.98
Sexual dysfunction (vs. no)	1.71	0.97–3.00	1.41	0.66–2.98
Venous thrombosis (vs. no)	1.44	0.45–4.63	1.31	0.28–6.17
Endometrial thickening (vs. no)	1.82	0.93–3.55	0.52	0.15–1.74
Eye toxicity (vs. no)	2.02	0.40–10.12	1.31	0.15–11.42
Hypertension (vs. no)	1.77	0.88–3.57	1.09	0.41–2.96
Dyslipidemia (vs. no)	1.48	0.58–3.77	0.76	0.17–3.37
Musculoskeletal symptoms (vs. no)	1.44	0.93–2.23	1.84*	1.02–3.31
Osteopenia (vs. no)	1.58	0.97–2.57	2.42*	1.31–4.48
Weight gain (vs. no)	1.42	0.89–2.27	1.10	0.57–2.12
Loss of hair (vs. no)	1.36	0.64–2.92	2.61*	1.10–6.21
Loss of appetite (vs. no)	1.85	0.90–3.84	2.38*	1.01–5.61
Fatigue (vs. no)	2.04*	1.31–3.18	2.69*	1.50–4.85

^†^Endocrine therapy; ^‡^aromatase inhibitor. *indicated a significant difference between the results of different groups.

### Multivariate Analysis of Depression and Anxiety

**FIGURE 1:**
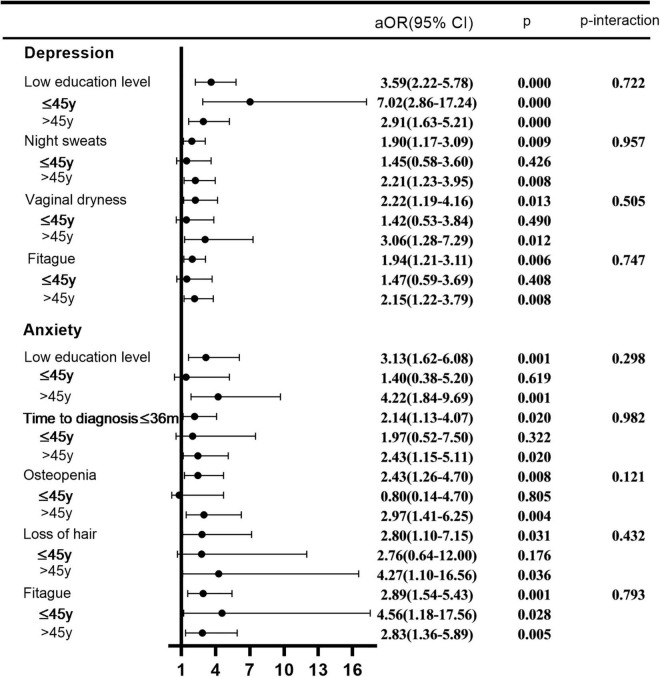
Multivariate analysis of depression and anxiety.

Figure 1 showed the results from multivariate analysis of depression and anxiety and their related factors. Multivariate model for depression included education level (≤ 8 years vs. > 8 years, OR = 3.59, 95% CI: 2.22–5.78), night sweats (yes vs. no, OR = 1.90, 95% CI: 1.17–3.09), vaginal dryness (yes vs. no, OR = 2.22, 95% CI: 1.19–4.16), and fatigue (yes vs. no, OR = 1.94, 95% CI: 1.21–3.11). Multivariate model for anxiety included education level (≤ 8 years vs. > 8 years, OR = 3.13, 95% CI: 1.62–6.08), time to diagnosis (≤ 3 years vs. > 3 years, OR = 2.14, 95% CI: 1.13–4.07), osteopenia (yes vs. no, OR = 2.43, 95% CI: 1.26–4.70), loss of hair (yes vs. no, OR = 2.80, 95% CI: 1.10–7.15), and fatigue (yes vs. no, OR = 2.89, 95% CI: 1.54–5.43).

Patients were stratified according to age (≤ 45 years and > 45 years) and a stratified analysis was performed as an exploratory. None of factor-age interactions was statistically significant.

## Discussion

Breast cancer patients in remission from cancer after therapy (surgery, chemotherapy and radiotherapy) are defined as cancer survivors, but they remain to endure psychological distress (Pitman et al., 2018). In particular, patients treated with endocrine treatment might live with side effects of ET and vulnerable to somatic symptoms. These factors are responsible for depression and anxiety observed in breast cancer patients. In this cross-sectional study, participants frequently experienced depression symptoms (33.4%), although anxiety was less common (13.3%). These women should receive support for their psychological adjustment during endocrine treatment.

The depression rate in the present study was in agreement with a previous similar South Africa study (36.6%) (Kagee et al., 2018). However, the prevalence in our study was significantly lower than a study conducted in Iran which reported the prevalence of depression among cancer patients to be 95.9% (Shakeri et al., 2016), the explanation for such a big difference could be the different living environments and medical conditions. High prevalence of depression was also reported from Turkey (46%) (Bener et al., 2017), Mexico (43%) (Pérez-Fortis et al., 2017), Qatar (47.2%) (Bener et al., 2017), and Nigeria (40.3%) (Popoola and Adewuya, 2012). Notably, depression prevalence in our study was higher than studies conducted in Ethiopia (25%) (Wondimagegnehu et al., 2019), Malaysia (22.0%) (Hassan et al., 2015), Levent (24.7%) (Akel et al., 2017), and Morocco (26.9%) (Berhili et al., 2017). The various depression prevalence rates reflect the differences in different demographic groups. Different tools used in studies, including Beck Depression Inventory (BDI) tool, Hospital Anxiety and Depression (HADs) tool, Center for Epidemiologic Studies Depression Scale (CESD) and Mini International Neuropsychiatric Interview (MINI), might also have contributed to this disparity.

Thoughts about death and recurrence of illness often disrupt patients’ focus, decision making, and sleep, showing different levels of anxiety. In a recent meta-analysis on breast cancer patients, the prevalence of anxiety among breast cancer patients was 41.9% (CI: 95%: 30.7–53.2%) (Hashemi et al., 2020). In the present study, the prevalence of anxiety in breast cancer patients accepting ET was 13.3%. Differences in the tools used to measure anxiety may lead to a wide range of anxiety prevalence among breast cancer patients (Hashemi et al., 2020). Although there are several tools for measuring anxiety, such as the Hospital Anxiety and Depression Scale (HADS), socioeconomic development index countries (SDI), the State-Trait Anxiety Inventory Questionnaire (STAI), they are lack specificity to specific clinical symptoms of breast cancer patients (Trask et al., 2000). Another reason could be the different study populations. Stage of the disease, the amount of family support, the patient’s economic level, their cultural context, level of education, residential status, other chronic medical illnesses, a history of psychiatric disorders, and perceived functioning limitations and demographic characteristics impact the level of anxiety in breast cancer patients (Stark et al., 2002; Pitman et al., 2018).

In totally, the different prevalence rates of anxiety and depression in different studies are due to the following reasons: (1) using different measurement tools, (2) the differences in the disease, socioeconomic and demographic factors. The prevalence of mental disorders among breast cancer patients was relatively high, which will have an adverse impact on patient’s adherence to treatment, quality of life and overall survival. This phenomenon should trigger our attention.

A significantly related factor to depression and anxiety was education level. Our finding reinforces the conclusion from the existing literature (Tsaras et al., 2018) and supports the conjecture that education can be a predictive factor in the occurrence of depression and anxiety among breast cancer patients dealing with the disease. This finding can be explained by the fact that women with higher educational levels can have better access to information regarding their health condition and they can fully understand the treatment plan and prognosis.

We found fatigue was strongly associated with mental disorders, which was consistent with the found of Lueboonthavatchai (2007). Cancer survivors have reported more frequent fatigue than their counterparts without a history of cancer (Bower et al., 2000). In cancer patients, fatigue constitutes a very distressing symptom that characteristically does not relieve with sleep (Fabi et al., 2020). A previous study showed fatigue might be more common among breast cancer survivors than among other cancer survivors and play an important role in survival (Matias et al., 2019). However, it is well known to be underdiagnosed and undertreated by clinic workers (Bower, 2014). Prior research found fatigue is mainly associated with anxiety, depression, distress, and sleep (Berger et al., 2012; Jones et al., 2016). In addition, endocrine therapy is a well-known trigger to fatigue (Franzoi et al., 2021). Fatigue is usually diagnosed by diagnosis tools, such as Fatigue Symptom Inventory (FSI), Cancer Fatigue Scale (CFS), and Fatigue Questionnaire (FQ). However, the difference in the use of diagnostic tools, and/or diagnostic criteria could lead to various prevalence rates (Krebber et al., 2014). Except for fatigue, we also found that other side effects of ET were related to depression and anxiety. However, further studies are warranted to verify our results.

It is noteworthy that patients have not received adequate attention from themselves. Only half of patients suffering from depression or anxiety seek help from their relatives and less than 6% of patients seek help from a psychiatrist. The *status quo* demands immediate attention from clinical workers.

### Study Limitations

This study had some limitations. First, it was a cross-sectional investigation, which could not reflect the factors associated with depression and anxiety in each period. Second, data were collected via telephone interviews, so the patience of the interviewee may affect the reliability. Third, side effects of ET included physical and psychological symptoms, some of which resemble the symptom of anxiety and depression. Fourth, economic status was lacking, which was meaningful factor in some previous studies. In addition, as already mentioned, body image issues, identity construction, social and intimate relationships are potential factors, but these factors were not incorporated in the present study, which may cause some bias. Finally, fatigue in our study was subjective patient-reported sensation rather than tool diagnosed, so the actual incidence might be overestimated. However, this study is meaningful because it provided useful guidance for clinical workers by identifying factors related to anxiety and depression in breast cancer patients accepting ET.

## Conclusion

Our study results confirmed that side effects of ET were significantly associated with anxiety and depression. Based on our results, more emphasis should be paid on minimizing the drugs side effects and clinical interventions are needed to reduce anxiety and depression in breast cancer patients accepting ET.

## Data Availability Statement

The raw data supporting the conclusions of this article will be made available by the authors, without undue reservation.

## Ethics Statement

The studies involving human participants were reviewed and approved by the Shanxi Bethune Hospital Ethics Committee. The patients/participants provided their written informed consent to participate in this study.

## Author Contributions

JG, RZ, and HL: conceptualization. JG: project administration. RZ: methodology and formal analysis. HL: data curation. JG and RZ: manuscript preparation. All authors approved the final manuscript.

## Conflict of Interest

The authors declare that the research was conducted in the absence of any commercial or financial relationships that could be construed as a potential conflict of interest.

## Publisher’s Note

All claims expressed in this article are solely those of the authors and do not necessarily represent those of their affiliated organizations, or those of the publisher, the editors and the reviewers. Any product that may be evaluated in this article, or claim that may be made by its manufacturer, is not guaranteed or endorsed by the publisher.
